# Therapeutic effects of three-strain probiotic combination on slow transit constipation: mechanistic insights into MAPK signaling pathway and gut microbiota restoration

**DOI:** 10.3389/fphar.2025.1684442

**Published:** 2025-10-20

**Authors:** Liya Liu, Peiyao Li, Youqin Chen, Guangqing Yang, Ying Cheng, Sijia Liu, Xinran Zhang, Yulun Wu, Qihong Liu, Peilin Zhao, Wenyi Fang, Yan Ren, Lunan Hu, Yanmin Liu, Kangning Li, Zhangran Chen, Xiao Ke, Qingquan Li, Aling Shen

**Affiliations:** ^1^ Academy of Integrative Medicine, Fujian University of Traditional Chinese Medicine, Fuzhou, China; ^2^ Fujian Key Laboratory of Integrative Medicine in Geriatrics, Fujian University of Traditional Chinese Medicine, Fuzhou, China; ^3^ Clinical Research Institute, The Second Affiliated Hospital of Traditional Chinese Medicine, Fuzhou, China; ^4^ Affiliated Sanming Integrated Medicine Hospital of Fujian University of Traditional Chinese Medicine, Sanming, China; ^5^ Department of Pediatrics, Case Western Reserve University School of Medicine, Rainbow Babies and Children’s Hospital, Cleveland, United States; ^6^ Department of Gastroenterology, The Second People’s Hospital Affiliated to Fujian University of Traditional Chinese Medicine, Fuzhou, China; ^7^ Fujian Clinical Medical Research Centre of Chinese Medicine for Spleen and Stomach, Fuzhou, China; ^8^ Shenzhen Wedge Microbiology Research Co., Ltd., Shenzhen, China; ^9^ Department of Pharmacology, School of Pharmacy, Fudan University, Shanghai, China

**Keywords:** three-strain probiotic combination, golden bifid, slow transit constipation, RNA sequencing, gut microbiota, MAPK signaling pathway

## Abstract

Three-Strain Probiotic Combination (Golden Bifid), a probiotic formulation composed of *Bifidobacterium longum*, *Lactobacillus bulgaricus*, and *Streptococcus thermophilus*, is widely used to modulate gut microbiota homeostasis and treat various gastrointestinal disorders. However, the specific molecular mechanisms underlying its therapeutic effects in slow transit constipation (STC) remain incompletely understood. In this study, we demonstrated that Golden Bifid alleviates loperamide-induced constipation by coordinately modulating host transcriptomic profiles, particularly the MAPK and serotonin signaling pathways, and restoring gut microbiota composition and diversity. These multi-omics findings provide novel mechanistic insights into the clinical efficacy of this probiotic combination, which have not been previously elucidated. Using a loperamide (LOP)-induced STC rat model, Golden Bifid was shown significantly increase defecation frequency, fecal water content, and intestinal motility, while improving the pathological damage of colonic tissues. It also elevated the protein expression of c-kit, 5-HT, 5-HT3R, and 5-HT4R in colonic tissue. RNA sequencing identified 1,998 differentially expressed transcripts in Golden Bifid group compared with the LOP group, with 899 upregulated and 1,099 downregulated. These transcripts were enriched in pathways, such as the mitogen-activated protein kinase (MAPK), tumor necrosis factor (TNF) and estrogen signaling pathway. Additionally, 16S rDNA sequencing demonstrated that the Golden Bifid partially restored gut microbiota structure, increased microbial diversity, and reversed the dysbiosis induced by LOP, notably reducing the abundance of *Patescibacteria* and modulating microbial taxa at both the phylum and genus levels to resemble the gut microbiota composition of the control group. These findings suggest that Golden Bifid alleviate STC by enhancing c-kit and 5-HT signaling, modulating the MAPK signaling pathway and pathway and restoring gut microbiota balance, offering promising therapeutic potential for STC treatment.

## 1 Introduction

Slow transit constipation (STC) is a common subtype of functional constipation, characterized by decreased intestinal motility, which delays the passage of feces through the colon, leading to constipation. Clinically, STC presents with symptoms such as dry stools, prolonged intervals between bowel movements, and difficulty in defecation (Bharucha and Lacy, 2020). Studies estimate that the prevalence of constipation ranges from 2.0% to 17.0% in China, 20% in the United States, and 13% in the United Kingdom (Black and Ford, 2018). In recent years, the changes in dietary habits, lifestyle, and the global aging population have contributed to, the increasing incidence of STC (Barberio et al., 2021). This rise in STC cases has had a profound impact on patients’ physical and mental health, and has increased the financial burden on healthcare systems. Although laxatives such as lactulose, macrogol (polyethylene glycol), magnesium sulfate, and prucalopride are widely used in clinical practice to alleviate constipation, recurrence of symptoms is common after discontinuation of these medications. Therefore, finding effective strategies for the prevention and treatment of STC, including developing suitable pharmacotherapies, is crucial for improving patients’ quality of life and reducing healthcare cost.

The pathogenesis of STC is complex and multifactorial, involving enteric nervous system (ENS) dysfunction, abnormalities in the quantity and function of interstitial cells of Cajal (ICC), altered levels of neurotransmitter and hormones such as serotonin (5-HT), gut microbiota dysbiosis, abnormal intestinal smooth muscle contraction, genetic factors, and environmental and lifestyle influences. ICCs, located in the gastrointestinal tract, generate and propagate slow-wave electrical activity, playing a critical role in transmitting neural signals to smooth muscle cells in the gut (Foong et al., 2020). Research indicates that ICCs in the colon regulate rhythmic colonic motility, and damage to or a reduction in the number of ICCs can result in impaired colonic motility, prolonged transit time, and difficulty in defecation. These mechanisms may contribute to the development of STC (Huizinga et al., 2021; Fung et al., 2019). Previous studies have demonstrated reduced expression of c-kit, a marker for ICCs, in the colon of STC rat models compared to controls (Xu et al., 2021).

5-hydroxytryptamine (5-HT) is a key important neurotransmitter that regulates gastrointestinal motility and secretion. By binding to receptors in the enteric nervous system, 5-HT promotes intestinal smooth muscle contraction, facilitating the movement of food and waste through the intestines (Dickson et al., 2010). In STC patients, impairments in the synthesis, release, or receptor action of 5-HT may lead to reduced intestinal motility and constipation. Pharmacological agents targeting 5-HT receptors show therapeutic potential for the treatment of STC. For instance, some 5-HT receptor agonists have been used to enhance intestinal motility and relieve constipation symptoms (Luo et al., 2025).

The gut microbiota is essential for maintaining gastrointestinal homeostasis, participating in various physiological processes, including food digestion, vitamin synthesis, bile acid transport, immune regulation, and epithelial cell metabolism (Huang et al., 2023). Disturbances in the gut microbiota can disturb intestinal homeostasis, potentially contributing to the development of constipation (Zheng et al., 2025).

Recent studies have shown that STC patients often exhibit reduced gut microbiota diversity and an imbalance specific bacterial species, with a decrease in beneficial bacteria, like *lactobacillus* and *prevotella*, and an increase in pathogenic bacteria, which may further weaken intestinal motility and exacerbate constipation (Malaguarnera et al., 2012; Ge et al., 2017). Moreover, gut microbiota-derived metabolites, such as short-chain fatty acids (SCFAs), can enhance intestinal motility by activating receptors (e.g., GPR43) on enteroendocrine cells to promote 5-HT synthesis. However, SCFA levels are typically lower in STC patients, impairing normal gut motility (Bai et al., 2025; Buey et al., 2023). Therefore, regulating the gut microbiota and its metabolic products may provide novel therapeutic strategies for STC.

Probiotics have shown significant potential in STC treatment by modulating gut microbiota, metabolic products, and neurotransmitters, ultimately improving intestinal motility and relieving constipation symptoms. For example, *lactobacillus plantarum* has been shown to improve constipation in STC mice by regulating gut neurotransmitter and hormone levels, including increasing 5-HT levels to enhance intestinal motility (Huang et al., 2023; Chen et al., 2022). Probiotics are live microorganisms that, when administered orally, offer health benefits to the host by producing beneficial enzymes, organic acids, vitamins, and antimicrobial substances. Numerous studies have reported that probiotics can enhance host health by modulating the gut microbiota and have been used to treat various gastrointestinal disorder, including constipation, diarrhea, infections, inflammatory bowel disease, and colon cancer (Luo et al., 2022; Mitelmão et al., 2022). Three-Strain Probiotic Combination (Golden Bifid) are a probiotic formulation containing *Bifidobacterium longum*, *Lactobacillus bulgaricus*, and *Streptococcus thermophilus*. It is widely used in clinical practice to regulate the gut microbiota and alleviate symptoms associated with gut microbiota dysbiosis (Luo et al., 2022; Wang et al., 2024). Previous studies have reported that Golden Bifid improves gastrointestinal symptoms and depression in patients with diarrhea-predominant irritable bowel syndrome by modulating the gut microbiota (Luo et al., 2022; Wang et al., 2024). However, the therapeutic effects and potential mechanisms of Golden Bifid in treating STC have not been previously reported.

Therefore, this study is the first to use RNA sequencing and 16S rDNA sequencing to investigate the therapeutic effects of Golden Bifid on a loperamide-induced STC rat model and to elucidate underlying molecular mechanisms. This research aims to provide experimental and theoretical evidence for the clinical application of Golden Bifid in treating STC.

## 2 Materials and methods

### 2.1 Reagents

Antibody specific to c-kits (Cat. No. 18696-1-AP) was provided by Proteintech (Wuhan, Hubei, China). Antibody specific to 5-HT3R (Cat. No. 32946) was provided by Signalway Antibody (MD, United States). Antibodies specific to 5-HT (Cat. No. BS-1126R) and 5-HT4R (Cat. No. BS-2127R) were provided by Beijing Boaosen Biotechnology Co., Ltd. (BIOSS, Beijing, China). Antibodies specific to Phospho-Erk (Cat. No. 4370) and Erk (Cat. No. 4695) were provided by Cell Signaling Technology (Danvers, MA, United States).

### 2.2 Preparation of golden bifid

Three-Strain Probiotic Combination (Golden Bifid, containing 1 × 10^7^ CFU/g *Bifidobacterium Longum*, 1 × 10^6^ CFU/g *Lactobacillus bulgaricus* and 1 × 10^6^ CFU/g *Streptococcus thermophiles,* Cat. No. S19980004) were obtained from Inner Mongolia Shuangqi Pharmaceutical Co., Ltd. (Inner Mongolia, China). The tablets were crushed under sterile conditions and immediately dissolved in sterile saline (0.9% NaCl) for gavage administration. To preserve bacterial viability, the suspension was freshly prepared and administered within 30 min of preparation.

### 2.3 Preparation of loperamide hydrochloride and prucalopride succinate tablets

Loperamide hydrochloride (LOP; Cat. No. H10910085) was obtained from Xian Janssen Pharmaceutical Co., Ltd. (Xi’an, China) and dissolved in sterile saline (0.9% NaCl) to prepare a stock solution of 0.5 mg/mL. The solution was freshly prepared prior to each administration and delivered via oral gavage at a dose of 3 mg/kg body weight in a volume of 10 mL/kg. Prucalopride succinate tablets (PST, Cat. No. H20183482) were purchased from Jiangsu Hansoh Pharmaceutical Group Co., Ltd. (Jiangsu, China) and similarly dissolved in sterile saline (0.9% NaCl) to obtain a stock solution of 1 mg/mL. The solution was also freshly prepared before each administration and administered via oral gavage at a dose of 0.18 mg/kg body weight in a volume of 10 mL/kg. All drug preparations were conducted under sterile conditions and used within 2 h of preparation. Control groups received an equivalent volume of sterile saline (0.9% NaCl).

### 2.4 Animals

**FIGURE 1:**
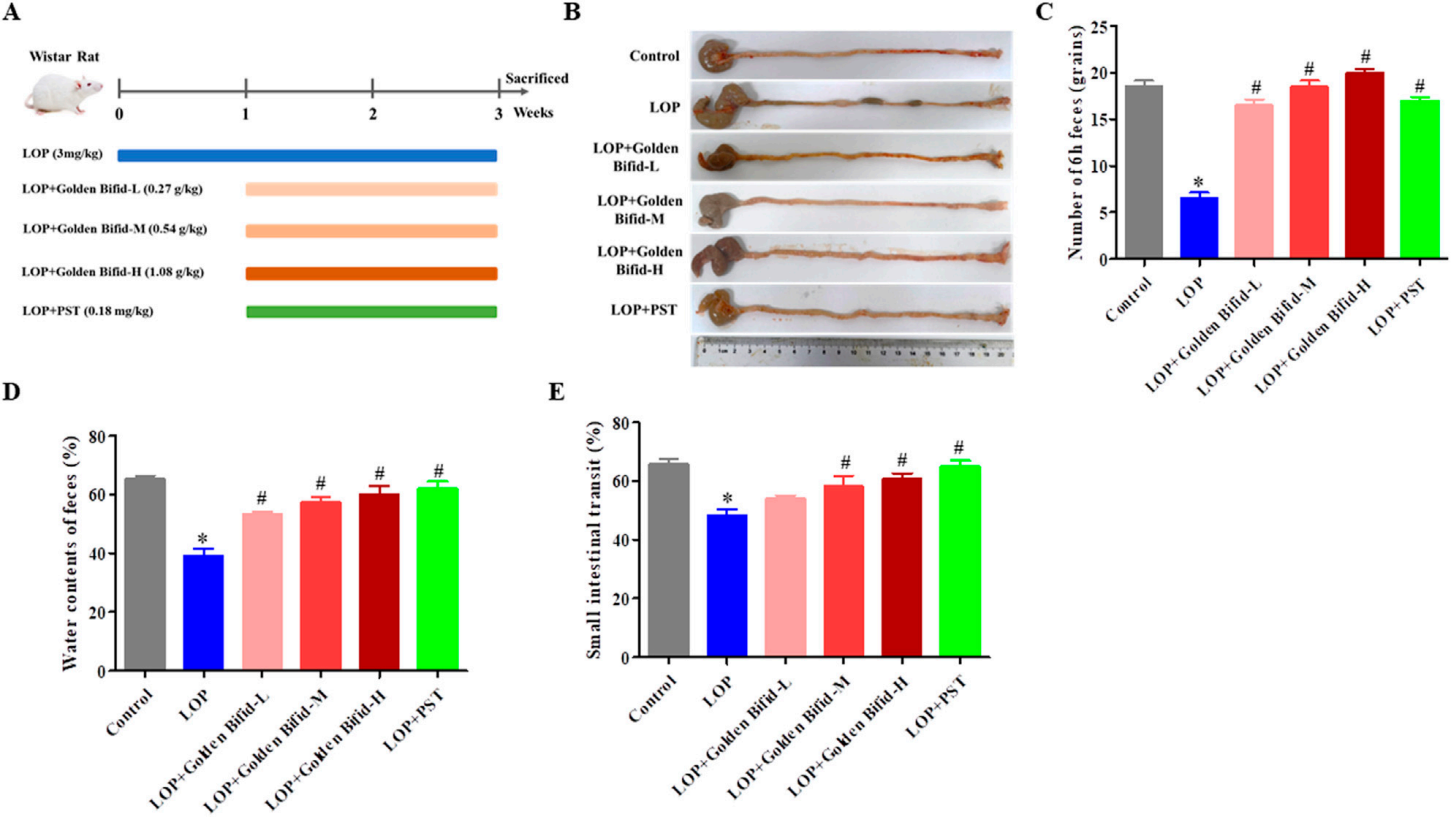
Effects of Golden Bifid on LOP-induced STC rats. **(A)** The experimental scheme for animal treatment; **(B)** Residual colon feces; **(C)** Number of feces in 6 h; **(D)** Fecal water content; **(E)** intestinal transit rate. *P < 0.05 vs. Control, #P < 0.05 vs. LOP.

Thirty-six SPF-grade male Wistar rats, aged 4–6 weeks, and weighing 150 ± 20 g, were purchased from Beijing Huafukang Bioscience Co., Ltd (Beijing, China). Animal License: SCXK (Beijing) 2019–0008. All rats were housed at the Experimental Animal Center of Fujian University of Traditional Chinese Medicine, under controlled conditions: temperature (20–22 °C), relative humidity (40%–60%), with a 12-hour light/dark cycle, and free access to food and water. This study protocol was approved by the Animal Ethics Committee of Fujian University of Traditional Chinese Medicine (Approval No.: FJTCM IACUC2022018). The rats were randomly divided into six groups: Control group, LOP group, LOP + low-dose Golden Bifid (LOP + Golden Bifid-L) group, LOP + medium-dose Golden Bifid (LOP + Golden Bifid-M) group, LOP + high-dose Golden Bifid (LOP + Golden Bifid-H) group, and LOP + Prucalopride succinate tablet (LOP + PST) group, with 6 rats in each group. The dosing regimen is illustrated in Figure 1A. Briefly, rats in the STC and treatment groups were administered loperamide (LOP; 3 mg/kg/day) by gavage, twice daily for 21 consecutive days to establish the STC model (Li et al., 2024; Yan et al., 2021). Starting on day 8, the Golden Bifid-L, Golden Bifid-M, and Golden Bifid-H groups were received different doses of Golden Bifid (L: 0.27, M: 0.54, and H:1.08 g/kg/d, respectively) via gavage 1 h after each loperamide administration, for 14 consecutive day. The medium dose (0.54 g/kg/d) was calculated using standard allometric scaling methods based on clinically recommended human doses. The PST group was administered prucalopride succinate tablets (0.18 mg/kg) via gavage in the same manner for 14 days. The Control and LOP groups were given an equivalent volume of sterile water (1 mL/100 g body weight) via gavage. One day before the end of treatment, fecal samples were collected from each rat, placed in sterile cryogenic tubes, and stored at −80 °C for 16S rDNA analysis.

### 2.5 Measurement of fecal water content

At the end of the treatment, each rat was placed in an individually metal cage, and fresh feces were collected every hour for 6 h. The wet weight of the feces was immediately recorded after collection. The feces were then dried at 60 °C for 6 h, and the fecal water content was calculated using the following formula: (wet weight of feces - dry weight of feces)/wet weight of feces × 100%.

### 2.6 Measurement of intestinal transit rate

The intestinal motility of the rats was assessed by measuring the movement of carbon ink through the intestines. Each rat was administered 3 mL of 10% activated charcoal via gavage 30 min after the final treatment. After 10 min, the rats were euthanized, and the small intestine, from the pylorus to the cecum was excised and measured. The intestinal transit rate (%) was calculated as the ratio of the distance traveled by the activated charcoal to the total length of the small intestine.

### 2.7 Hematoxylin and eosin (H&E) staining

Colonic tissues were fixed in 4% formaldehyde for 48 h, then dehydrated, embedded in paraffin, and sectioned into 4-μm slices. After deparaffinization, the sections were stained with hematoxylin and eosin to assess colonic tissue morphology, mucosal thickness and muscle thickness (Zeng et al., 2025).

### 2.8 Immunohistochemical (IHC) staining

After deparaffinization and dehydration of the 4-μm colonic tissue sections, heat-induced antigen retrieval was performed. The sections were then incubated overnight at 4 °C with primary antibodies against c-kit, 5-HT, 5-HT3R, 5-HT4R, Phospho-Erk and Erk (all dilution at 1:100). Afterward, the sections were incubated with a horseradish peroxidase (HRP)-conjugated secondary antibody, then a streptavidin-alkaline phosphatase complex. 3,3′-diaminobenzidine (DAB) was used for color development, and hematoxylin was applied as a counterstain. Under a light microscope (Leica, Wetzlar, Germany) at ×400 magnification, the slides were imaged and the percentage of positive expression from five fields was assessed using the ImageJ analysis system (Bethesda, MD).

### 2.9 RNA sequencing

The colonic tissues from the Control, LOP and Golden Bifid-M groups were stored in RNAlater (Takara, Beijing, China) at room temperature for 1 h and moved to −20 °C for long-time storage. Total RNA was extracted using the mirVana miRNA Isolation Kit (Thermo Fisher Scientific, Grand Island, NY, United States), according to the manufacturer’s protocol. RNA concentration and quality were evaluated with Qubit 3.0 and Agilent 2,100 Bioanalyzer, respectively. RNA samples with a RIN ≥7 were used for RNA sequencing. RNA sequencing (RNA-seq) library was constructed by CapitalBio Technology (Beijing, China). In brief, rRNA was removed from total RNA using Ribo-Zero Magnetic Kit, and the poly(A)-tailed mRNA molecules were generated using the NEBNext Ploy(A) mRNA Magnetic Isolation Module Kit (New England Biolabs, MA, United States). The libraries were quantified on Agilent 2,100 Bioanalyzer using KAPA Library Quantification Kit and subjected to paired-end sequencing on an Illumina Hiseq sequencer (Illumina). The raw data were processed through a bioinformatics pipeline, including quality assessment (FastQC v0.11.5), alignment (HISAT2 v2.1.0), genes and transcripts reconstruction (StringTie v1.3.3b), and differentially expressed transcripts analysis (DESeq v1.28.0). Functional annotations were performed using Kyoto Encyclopedia of Genes and Genomes (KEGG). We uploaded the raw data to the Gene Expression Omnibus (GEO) database (Submission No.: GSE273280).

### 2.10 Quantitative real-time reverse transcription polymerase chain reaction (qRT-PCR)

Total RNA was extracted from colon tissue using RNAiso Plus reagent (Takara, Beijing, China). The total RNA was quantified using NanoDrop 2000 (Thermo Fisher, United States). Reverse transcription into complementary DNA (cDNA) was performed according to the manufacturer’s instructions using the PrimeScript RT reagent kit (Takara). The cDNA was then amplified on a quantitative PCR instrument according to the TB Green^®^ Premix Ex Taq (Takara). The process of the PCR reaction was initiated at 95 °C for 30 s, followed by 40 cycles (95 °C for 5 s and 60 °C for 30 s). Relative mRNA levels were calculated using the comparative method (2^−ΔΔCT^), with GAPDH serving as an internal reference gene. The primer sequences (General Biol, China) used was listed in Supplementary Table S1.

### 2.11 Microbial 16S rDNA gene sequencing and analysis

Fecal samples were subjected to 16S rDNA sequencing (CapitalBio Technology, Beijing, China). Briefly, microbial DNA was extracted using a Magnetic Bead-based Soil and Fecal Genomic DNA Extraction Kit (Catalog No.: DP712, Tiangen Biotech, Beijing, China). DNA concentration and purity were assessed by 1% agarose gels electrophoresis to evaluate DNA integrity, and spectrophotometry to ensure an A260/A280 ratio >1.8. DNA samples were diluted to 1 ng/μL for PCR amplification. The V3-V4 regions of bacterial 16S rRNA genes were amplified using universal primers: forward 5′-CCTAYGGGRBGCASCAG-3′ and reverse 5′-GGACTACNNGGGTATCTAAT-3′, in conjunction with Phusion^®^ High-Fidelity PCR Master Mix (New England Biolabs, United States). PCR products were purified using Qiagen Gel Extraction Kit (Qiagen, Germany). Purified amplicons were used for library construction following standard Illumina 16S amplicon library preparation protocols, incorporating appropriate adapters and barcodes for sample multiplexing. Library quantification was performed using Qubit^®^ 2.0 Fluorometer (Thermo Fisher Scientific, United States) and qPCR. Paired-end sequencing (2 × 250 bp) was performed on the Illumina NovaSeq™ 6,000 platform (Illumina, United States). Raw paired-end reads were assembled using FLASH (V1.2.7) and subject to quality filtering using QIIME (V1.9.1). Chimeric sequences were identified and removal using the UCHIME algorithm by comparison against the Silva database. OTUs were clustered using UPARSE software (V7.0.1001), with sequences sharing ≥97% similarity assigned to the same OTU. Taxonomic annotation of representative OTU sequences was performed using the Silva Database via Mothur algorithm. Alpha diversity indices, including Shannon and Simpson indices, were calculated using QIIME (V1.7.0) to assess within-sample microbial diversity. Beta diversity was analyzed using both weighted and unweighted UniFrac distances in QIIME (V1.9.1), with downstream visualization performed via Principal Coordinates Analysis (PCoA) and UPGMA clustering to evaluate between-sample microbial community differences.

### 2.12 Statistical analysis

Statistical analysis in this study was performed using SPSS software version 26.0 (IBM Corp., Chicago, IL, United States). All quantitative data are expressed as mean ± standard deviation (SD). For normally distributed data, one-way analysis of variance (ANOVA) was used to compare differences among three or more groups; for homoscedastic data, the Bonferroni method was applied; otherwise, the Games-Howell test was used. For non-parametric data, the Kruskal–Wallis test was used. A P-value of <0.05 was considered statistically significant.

## 3 Results

### 3.1 Effects of golden bifid on clinical manifestations changes in STC rats

To explore the therapeutic effect of Golden Bifid on constipation, LOP-induced STC model rats were established, and different dosage Golden Bifid was orally administered. The timeline of model establishment and treatment is shown in Figure 1A. Compared with the control group, the LOP group had fewer, small, and hard stools in the colonic tissue and more cecal contents, while fecal content in the intestines was significantly reduced after treatment with different doses of Golden Bifid or PST (Figure 1B). Compared with the control group, the LOP group of rats exhibited a significant reduction in fecal water content and total fecal quantity within 6 h. However, after treatment with different doses of Golden Bifid or PST, both fecal water content and total fecal quantity were significantly increased (Figures 1C,D). Additionally, the intestinal transit rate in the LOP group was significantly reduced compared to the control group (Figure 1E), whereas treatment with different doses of Golden Bifid or PST significantly increased the intestinal transit rate.

**FIGURE 2:**
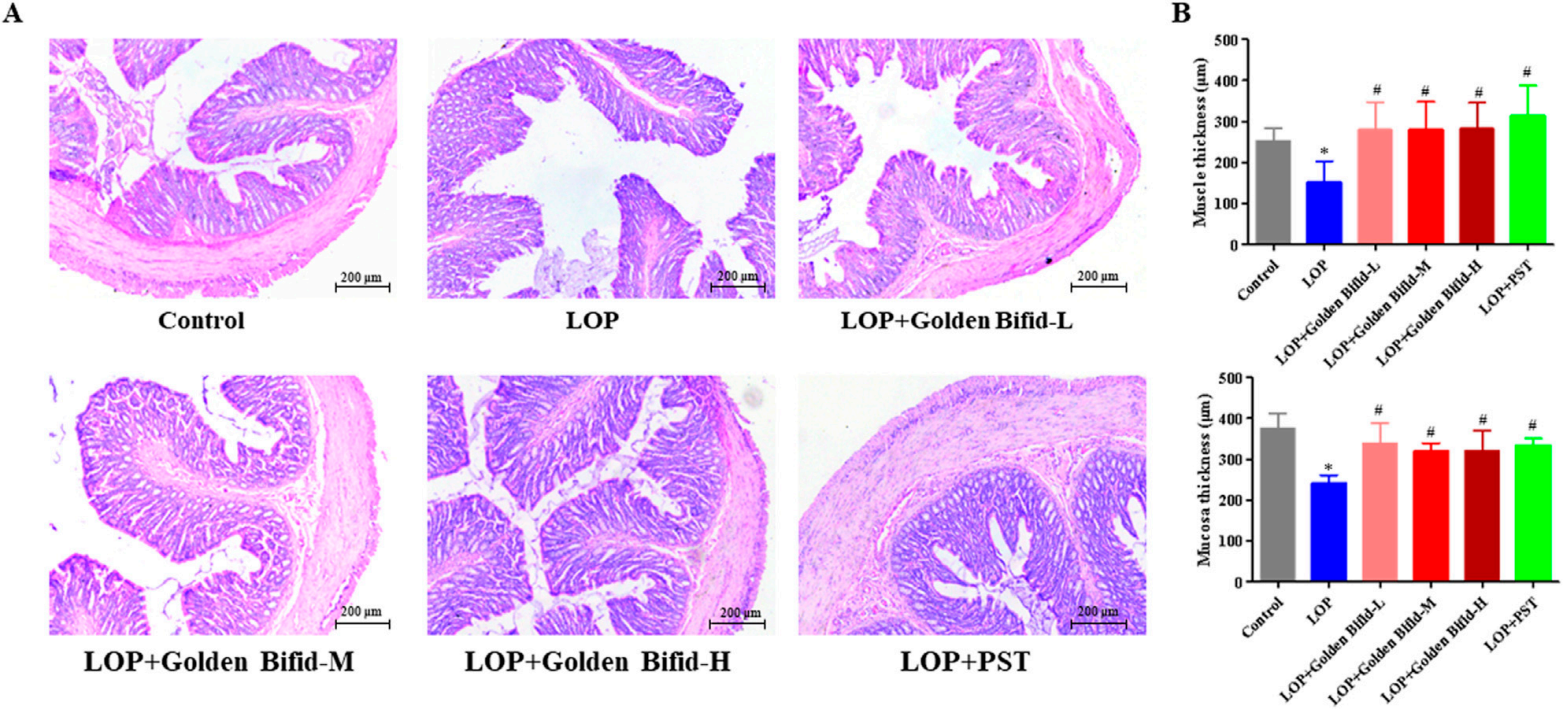
Effects of Golden Bifid on histopathological changes in LOP-induced STC rats. **(A)** The histopathological changes in colonic tissue were observed under a microscope following H&E staining. Representative images were taken at a magnification of ×100. **(B)** Tissue thickness of mucosal and muscular layers. *P < 0.05 vs. Control, #P < 0.05 vs. LOP.

H&E staining revealed that the colonic mucosa in the control group was intact, with a well-structured intestinal wall and no inflammatory cell infiltration. In contrast, the LOP group exhibited a disordered arrangement of intestinal glands, epithelial cell shedding, and dilated stromal blood vessels in the submucosa of the colon. However, after treatment with different doses of Golden Bifid or PST, the colonic mucosa and intestinal wall structure were significantly restored (Figure 2A). In addition, the thickness of the mucosal and muscle layers was measured. The analysis showed that, compared with the control group, tissue thickness was significantly reduced in the LOP group. These alterations were markedly reversed following treatment with different doses of Golden Bifid or PST (Figure 2B). These results suggest that Golden Bifid treatment significantly improves constipation in STC rats, showing effects comparable to the PST group.

### 3.2 Effects of golden bifid on the expression of c-kit protein in intestinal cells of cajal in STC rats

**FIGURE 3:**
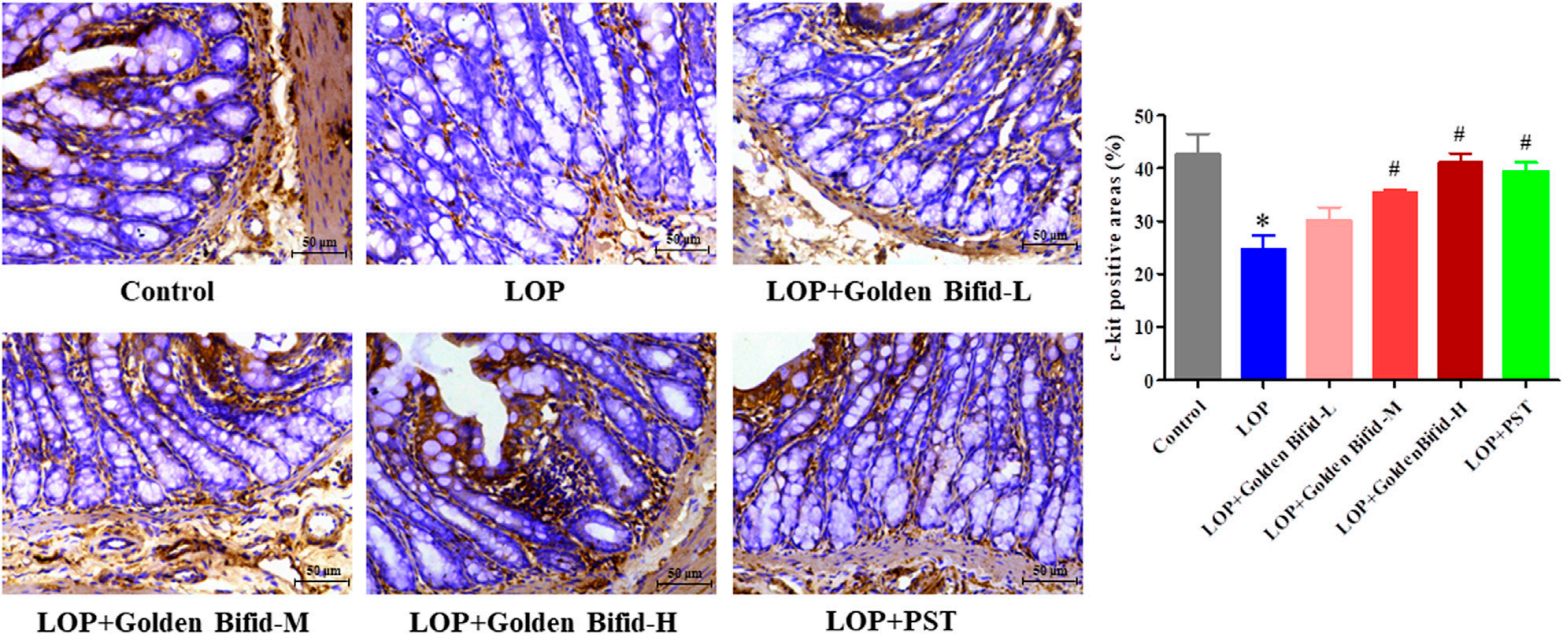
Effects of Golden Bifid on c-kit protein expression in LOP-induced STC rats. IHC was performed to detect c-kit expression in colon tissues. Representative images of IHC analysis were taken at a magnification of ×400,and staining intensity was analyzed. *P < 0.05 vs. Control, #P < 0.05 vs. LOP.

IHC analysis was performed to assess changes in intercellular connections within the intestinal mucosa by detecting c-kit protein expression. Compared with the control group, the LOP group induced a significant decrease in the expression of c-kit. However, after treatment with different doses of Golden Bifid or PST, the expression of c-kit significantly increased (Figure 3).

### 3.3 Effects of golden bifid on the expression of 5-HT and its receptors (5-HT3R, 5-HT4R) in the intestinal tissue of STC rats

**FIGURE 4:**
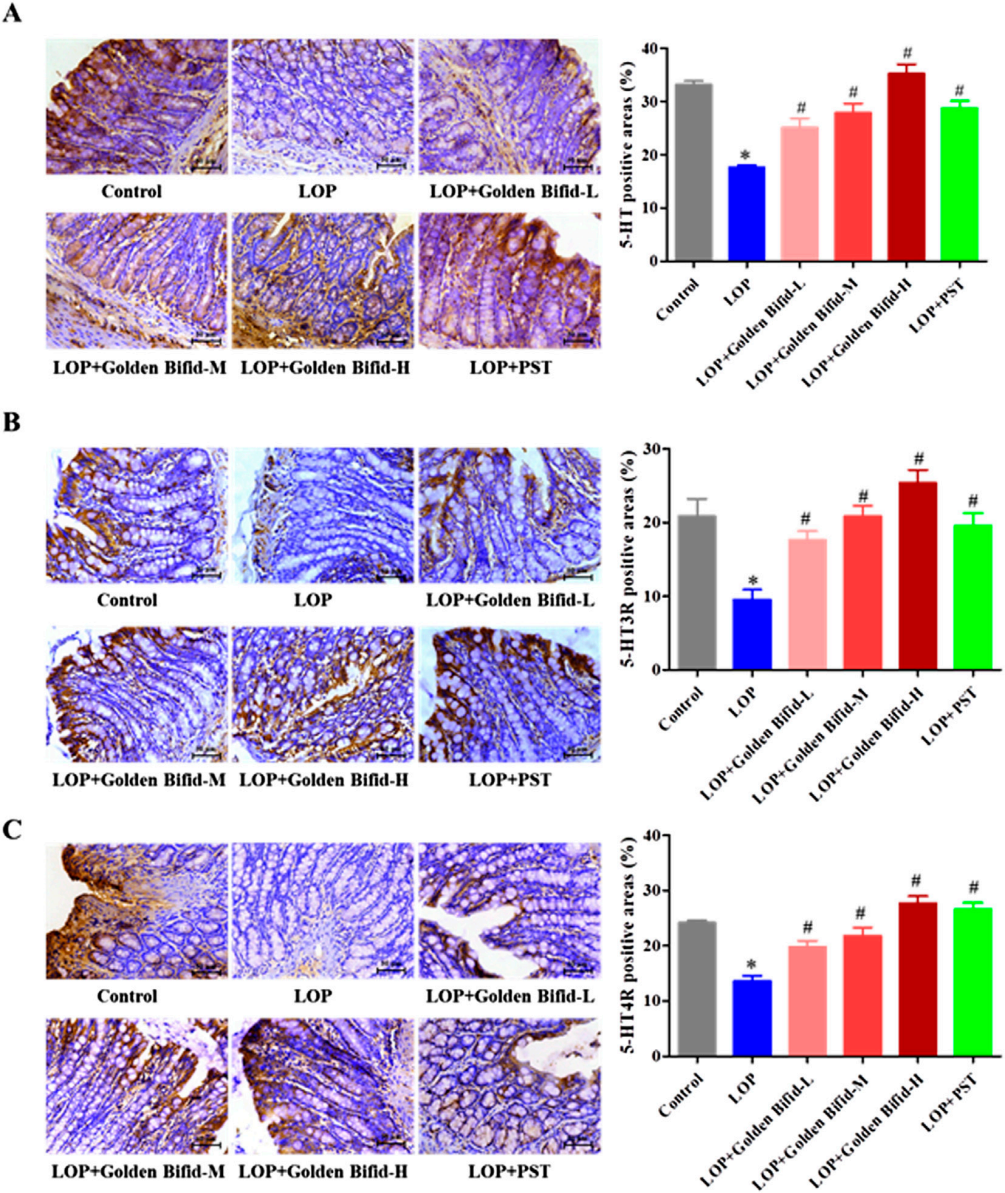
Effects of Golden Bifid on the expression of 5-HT and its receptors (5-HT3R, 5-HT4R) in LOP-induced STC rats. IHC was performed to detect the expression of **(A)** 5-HT, **(B)** 5-HT3R and **(C)** 5-HT4R in colon tissues. Representative images of IHC analysis were taken at a magnification of ×400, and staining intensity was analyzed. *P < 0.05 vs. Control, #P < 0.05 vs. LOP.

IHC analysis of 5-HT, 5-HT3R, and 5-HT4R expression in the colonic tissue of rats, as shown in Figure 4, revealed that the positive cell areas of 5-HT, 5-HT3R, and 5-HT4R were significantly decreased in the LOP group than in the control group. However, after treatment with different doses of Golden Bifid or PST, the expression of these proteins was significantly increased. These results suggest that Golden Bifid treatment restored the expression of c-kit and key components of 5-HT signaling pathway, which may contribute to enhance intestinal motility and improve neural-epithelial communication in the constipated colon.

### 3.4 Effects of golden bifid on differentially expressed transcripts and pathways in intestinal tissue of STC rats via RNA sequencing

**FIGURE 5:**
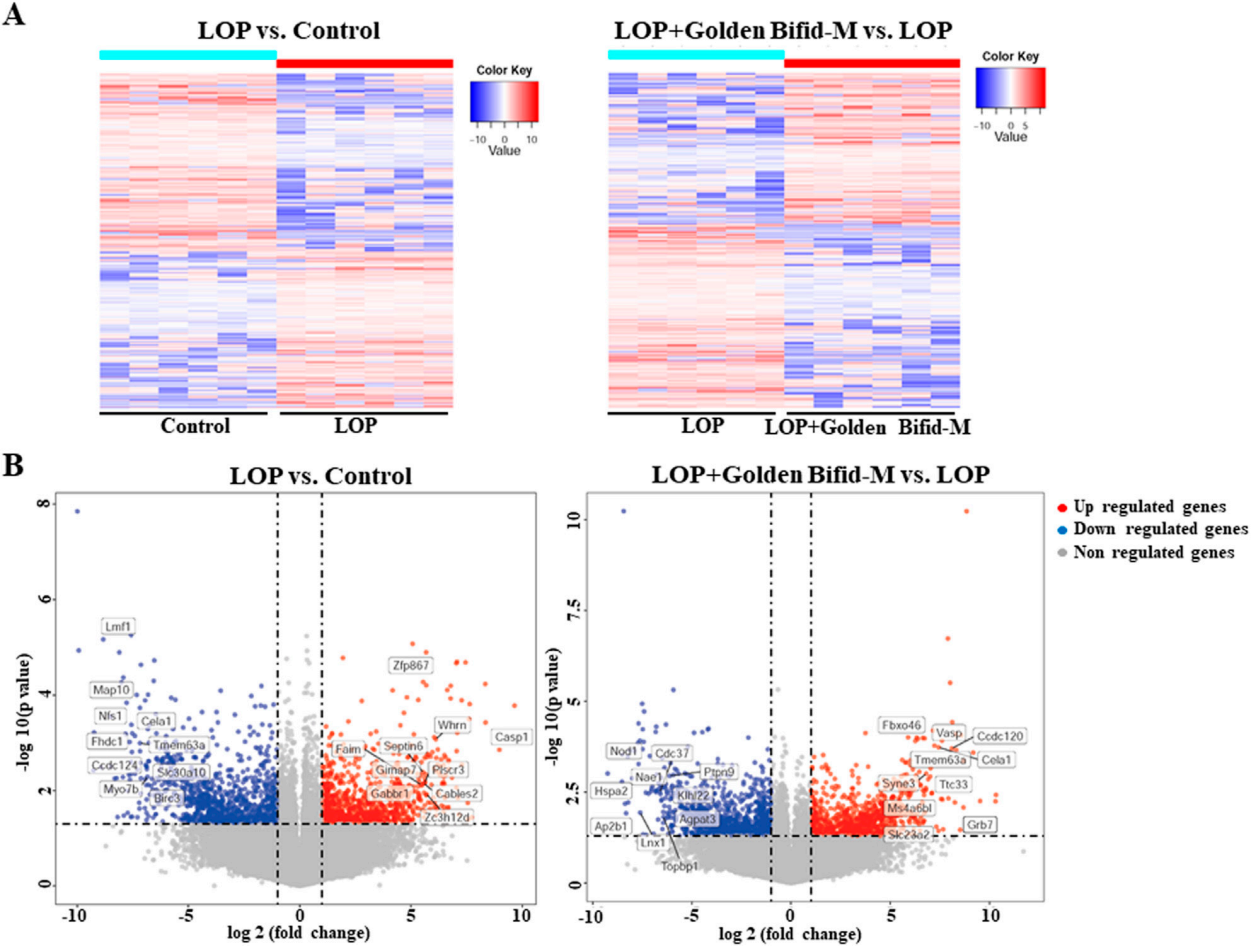
Genome-wide gene expression profiling in the colon tissues of rats. **(A)** Hierarchical clustering plots and **(B)** Volcano plots comparing gene expression profiles between groups (|fold change| ≥2, P < 0.05).

Since the medium-dose group showed the most significant improvements in key constipation-related phenotypes, tissue samples from this group were selected for RNA sequencing to further investigate the mechanisms underlying Golden Bifid effects on constipation. As shown in Figure 5, compared to the control group, the LOP group had 2,044 differentially expressed transcripts, with 957 upregulated and 1,087 downregulated. In comparison to the LOP group, the medium-dose Golden Bifid intervention resulted in 1,998 differentially expressed transcripts, with 899 upregulated and 1,099 downregulated.

**FIGURE 6:**
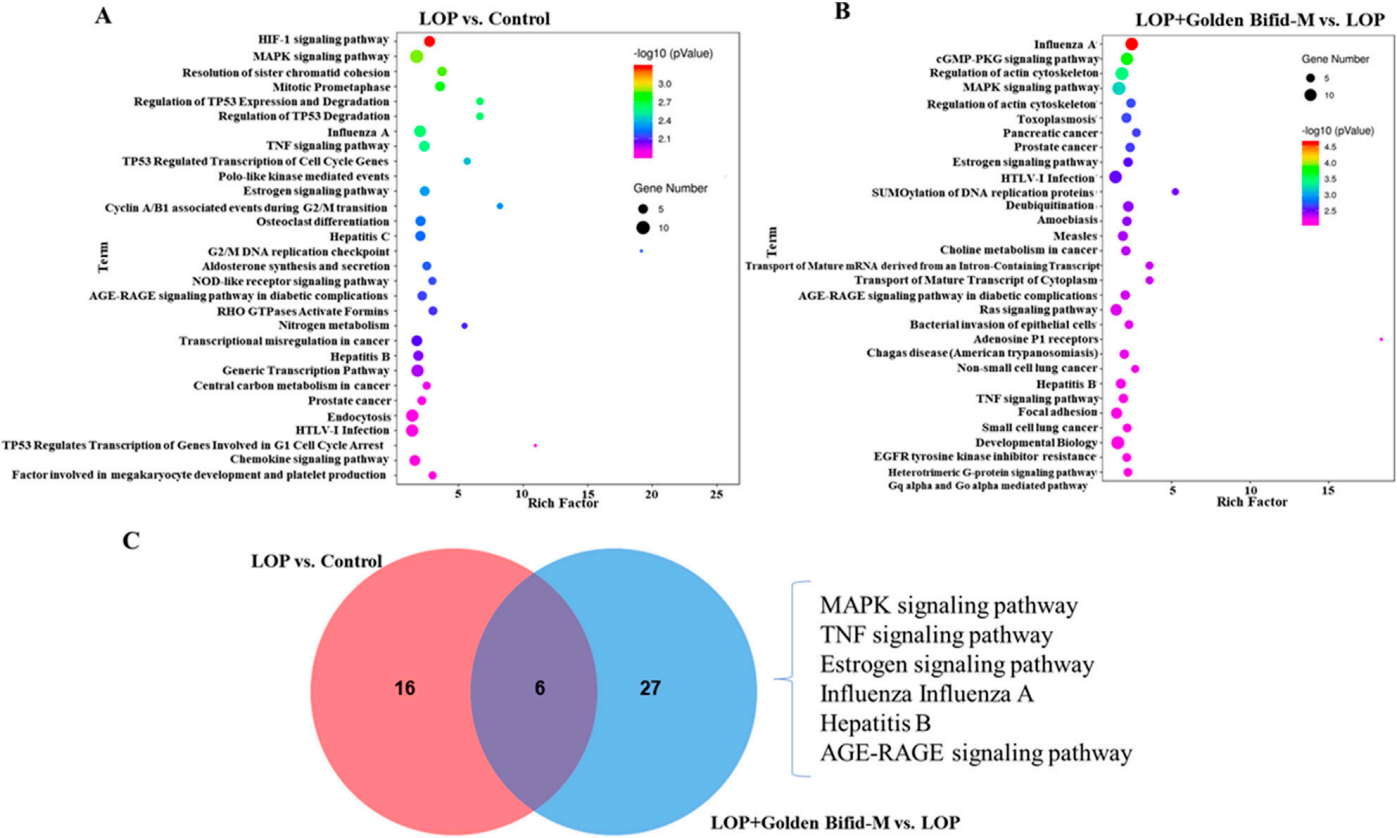
KEGG signaling pathways enrichment analysis in the colon tissue of rats. **(A,B)** KEGG pathway enrichment analysis of differentially expressed transcripts in the comparisons of LOP vs. Control and LOP + Golden Bifid-M vs. LOP. **(C)** Overlapping KEGG pathways between the two comparisons.

Further enrichment analysis was conducted on the differentially expressed transcripts in each group to identify potential pathways that may be regulated by Golden Bifid in inhibiting the development of STC. A P-value <0.01 was used as the screening criterion. The enrichment results are shown in Figure 6. A total of 22 pathways were effectively enriched in the differentially expressed transcripts between the LOP and control groups, mainly focusing on the MAPK, TNF signaling pathway and TP53 Regulates Transcription of Cell Cycle Genes, among other signaling pathways (Figure 6A; Supplementary Table S2). A total of 33 pathways were effectively enriched in the differentially expressed transcripts between the LOP + Golden Bifid-M and LOP groups, mainly focusing on the MAPK signaling pathway, Inflammation mediated by chemokine and cytokine signaling pathway, among others (Figure 6B; Supplementary Table S3). The intersection of the enriched pathways from both comparisons yielded 6 common pathways, including the MAPK signaling pathway, TNF signaling pathway, Estrogen signaling pathway, Influenza A, Hepatitis B and AGE-RAGE signaling pathway in diabetic complications (Figure 6C).

### 3.5 Validation of RNA sequencing results

**FIGURE 7:**
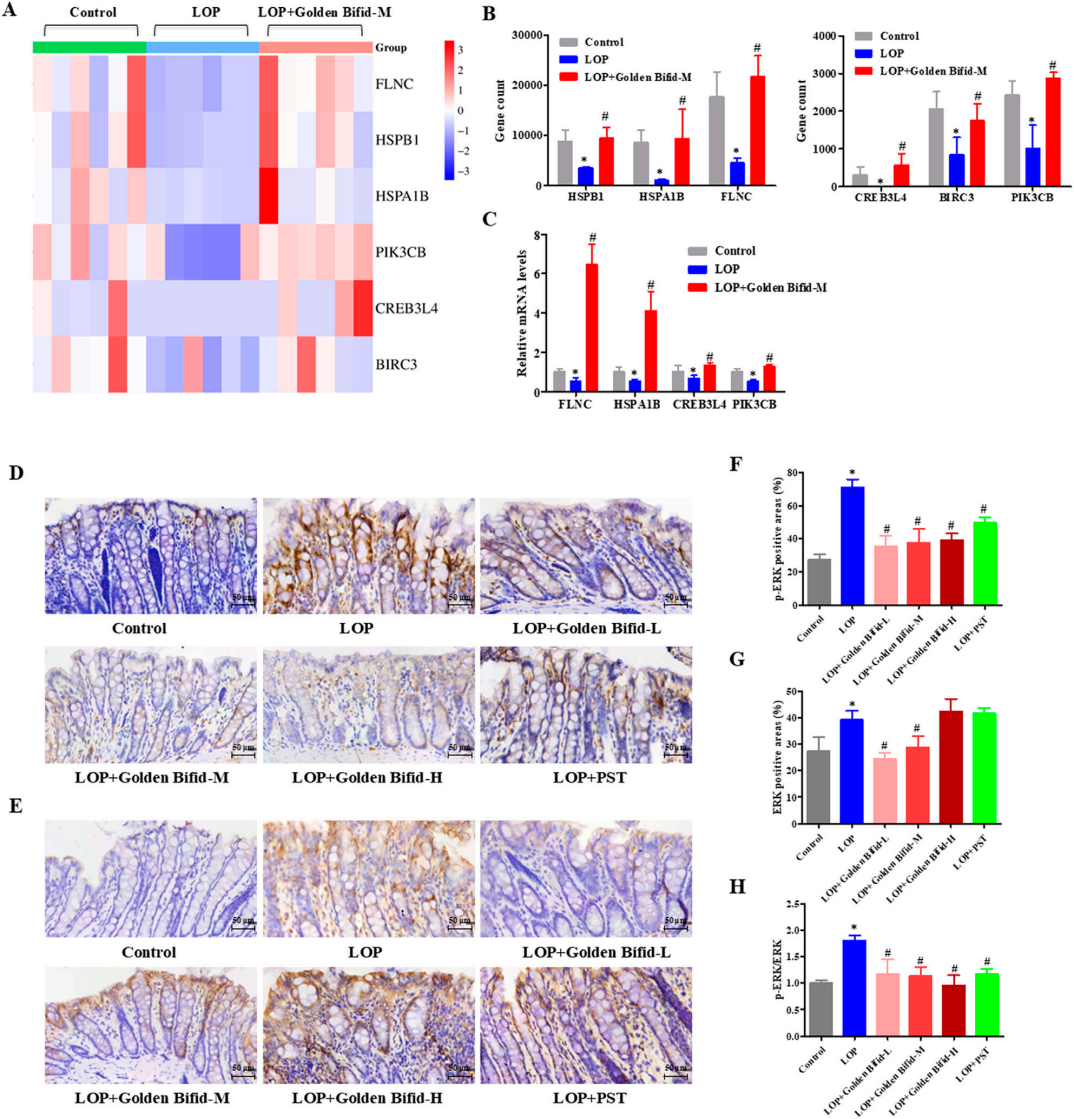
Effects of Golden Bifid on the MAPK Signaling pathway in LOP-induced STC rats. **(A)** RNA-seq heat map of MAPK-pathway genes; red/blue indicate up- or downregulation versus Control. **(B)** Column view of RNA-seq log2-fold changes for key MAPK genes. **(C)** qRT-PCR validation of selected transcripts. **(D,E)** IHC was performed to detect **(D)** p-ERK and **(E)** ERK expression in colon tissues. Representative images of IHC analysis were taken at a magnification of ×400. **(F,G)** Positive staining area of **(F)** p-ERK and **(G)** ERK were analyzed. **(H)** p-ERK/ERK ratio calculated from positive-area measurements. *P < 0.05 vs Control, #P < 0.05 vs LOP.

The resulting transcriptomic data revealed significant enrichment of the MAPK and TNF signaling pathway. To ensure experimental consistency and data coherence, we validated the expression of genes-related to these pathways in the same medium-dose group using qRT-PCR. Additionally, IHC analysis was used to detect the expression of phosphorylated proteins involved in these key signaling pathways. The results indicated that compared with the control group, the mRNA expression of FLNC, HSPB1, HSPA1B, PIK3CB, CREB3L4, and BIRC3 was significantly downregulated in the LOP group, while the medium-dose Golden Bifid intervention significantly upregulated their expression (Figures 7B,C). These findings were consistent with the RNA sequencing results (Figure 7A). IHC analysis of phosphorylated ERK, a key protein in the MAPK pathway, revealed that p-ERK expression was significantly elevated in the LOP group compared to the control group, while the medium-dose Golden Bifid and PST treatment significantly decreased p-ERK expression (Figures 7D–H). These results suggest that Golden Bifid treatment inhibit the activation of MAPK signaling pathways.

### 3.6 Effects of golden bifid on colonic microbial communities in intestinal tissue of STC rats

**FIGURE 8:**
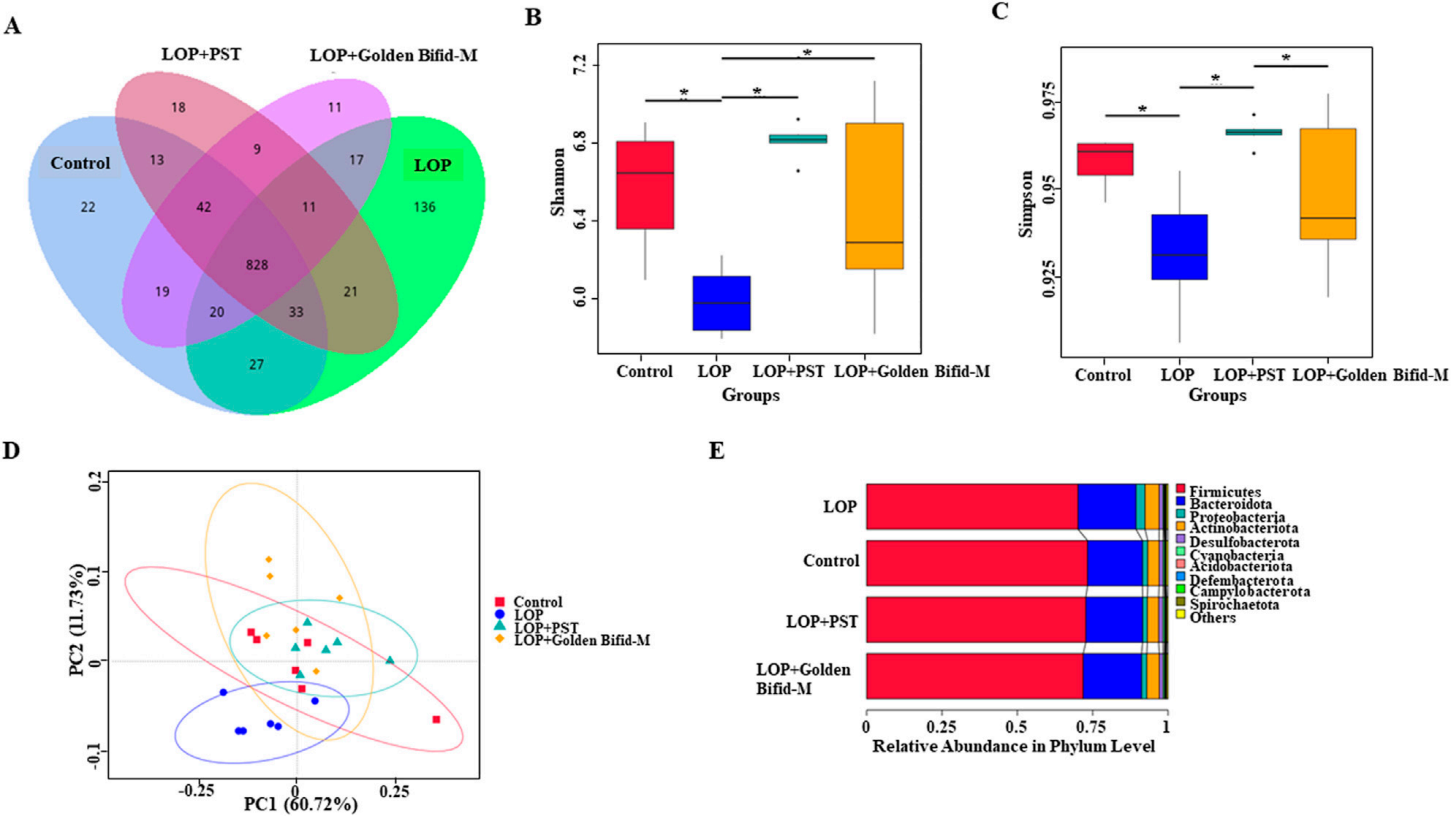
Effects of Golden Bifid on gut microbiota in LOP-induced STC rats. **(A)** Venn diagram of OTUs. **(B,C)** α diversity analysis (Shannon and Simpson indexes). **(D)** β diversity analysis (PCoA analysis). **(E)** β diversity analysis (UPGMA clustering). *P < 0.05.

To investigate the effects of Golden Bifid on colonic microbial communities in rats; 16S rDNA sequencing was used to analyze the colonic microbiota of each group. Venn diagram analysis identified a total of 1,227 operational taxonomic units (OTUs) detected across the four groups, with 1,004 OTUs in the control group, 1,093 in the LOP group, 957 in the Golden Bifid-M group, and 975 in the PST group. The analysis of characteristic OTUs revealed 22 OTUs unique to the Control group, 136 unique to the LOP group, 11 unique to the Golden Bifid-M group, and 18 unique to the PST group (Figure 8A). The composition of colonic microbial communities in the LOP group differed significantly from the control group, but Golden Bifid-M and PST treatment partially restored the microbial composition in STC rats. These results suggest that Golden Bifid treatment regulate the composition of colonic microbial communities in STC rats.

### 3.7 Diversity analysis of golden bifid on colonic microbial communities in intestinal tissue of STC rats

Compared with the control group, the Shannon and Simpson indices were significantly lower in the LOP group, but both indices were significantly increased following Golden Bifid-M or PST intervention (Figures 8B,C). These results suggest that colonic microbial α-diversity in STC rats improved following Golden Bifid-M or PST intervention. Additionally, β-diversity analysis revealed significant differences in fecal microbial communities between the LOP group and the Golden Bifid-M and PST groups, with greater similarity between the Golden Bifid-M, PST, and control groups (Figure 8D). UPGMA analysis showed that the samples in this study were roughly clustered into 4 clusters (Figure 8E), which were generally consistent with the results of sample grouping, and PCoA analysis. The microbial diversity analysis further confirmed that Golden Bifid positively regulates the recovery of colonic microbial communities in STC rats.

### 3.8 Overview of microbial community composition of golden bifid on colonic microbial communities in intestinal tissue of STC rats

**FIGURE 9:**
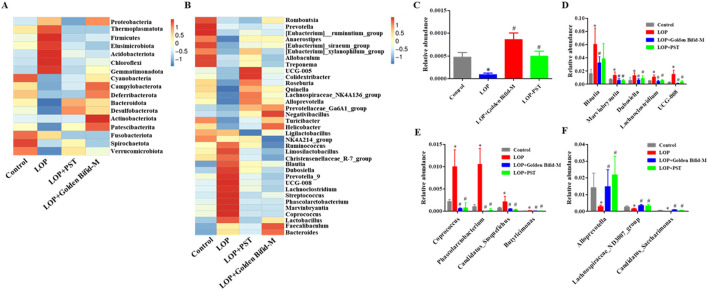
Difference in gut microbiota at the Phylum and Genus levels. **(A)** Heatmap of relative abundance of the top 10 bacteria genera at the Phylum levels. **(B)** Heatmap of relative abundance of the top 35 bacteria genera at the Genus levels. **(C)** Relative abundances of *Patescibacteria* at the Phylum levels. **(D‐F)** Relative abundances of *Blautia, Dubosiella, UCG-008, Lachnoclostridium, Phascolarctobacterium, Marvinbryantia, Candidatus_Stoquefichus, Butyricimonas*, *Coprococcus, Lachnospiraceae_ND3007_group, Alloprevotell* and *Candidatus_Saccharimonas* at the Genus levels. *P < 0.05, vs. Control, #P < 0.05, vs. LOP.

The composition of gut microbial communities was analyzed at different taxonomic levels. At the phylum level, the top four dominant bacterial groups in the rat colon were *Firmicutes, Bacteroidota, Spirochaetota,* and *Desulfobacterota*. In the LOP-induced STC rats, *Firmicutes* expanded significantly, while *Bacteroidota* contracted. After treatment with Golden Bifid and PST, the relative abundance of *Firmicutes* and *Bacteroidota* was restored to levels similar to those in the control group (Figure 9A). Notably, the relative abundance of *Patescibacteria* was significantly reduced in the LOP group compared to the control group, but this reduction was restored after Golden Bifid treatment (Figure 9C). Additionally, Golden Bifid treatment significantly improved the proportion of *Patescibacteria* compared to the LOP group.

At the genus level, Golden Bifid treatment reversed LOP-induced dysbiosis, significantly reducing the relative abundance of *Blautia, Dubosiella, UCG-008, Lachnoclostridium, Phascolarctobacterium, Marvinbryantia, Candidatus_Stoquefichus, Butyricimonas* and *Coprococcus* restoring them to the control group levels (Figure 9B). Golden Bifid treatment also increased the relative abundance of *Lachnospiraceae_ND3007_group*, *Alloprevotell* and *Candidatus_Saccharimonas* (Figures 9D–F). These results indicate that Golden Bifid treatment restores gut microbiota composition at various taxonomic levels, reversing dysbiosis caused by LOP-induced STC.

## 4 Discussion

Slow transit constipation (STC) is a disorder characterized by impaired colonic transit function, leading to reduced defecation frequency and increased stool hardness. The incidence of STC has been increasing annually due to various factors such as psychological stress, dietary changes, and environmental shifts (Bharucha and Lacy, 2020). Severe cases of STC can cause intestinal mucosal damage and increased gut permeability, contributing to the development of various diseases and significantly impacting overall health. In this study, we established an STC rat model using the well-validated method of loperamide (LOP) administration. The model displayed typical STC characteristics, including reduced defecation frequency, dry stools, delayed intestinal transit, and severe colonic damage (Ohkusa et al., 2019). Treatment with Three-Strain Probiotic Combination (Golden Bifid) improved defecation frequency, increased stool water content, restored small intestinal transit, and alleviated colonic tissue damage in STC rats, further confirmed its therapeutic potential in managing STC.

Previous studies have identified damage to interstitial cells of ICC cells as a key mechanism in STC pathogenesis (Xu et al., 2021). ICCs essential for gastrointestinal motility, as they mediate communication between the enteric nerves are us system and smooth muscle cells. A reduction in ICC number or function can impair smooth muscle contraction, leading to colonic motility disorders and delayed intestinal transit (Kondo et al., 2015; Sanders et al., 2016). The c-kit protein is a marker of ICCs and plays a crucial role in their growth and maintenance (Tan et al., 2014). Consistent with previous reports, our study found reduced expression of c-kit in the colonic tissue of STC rats. However, Golden Bifid treatment at various doses significantly upregulated c-kit expression, like the effects seen in the positive control group. These findings suggest that Golden Bifid may alleviate STC by promoting ICC function and maintaining normal colonic motility.

5-HT is a vital neurotransmitter that stimulates gastrointestinal motility and by promoting colonic smooth muscle contraction and activating cholinergic pathways (Taniyama et al., 2000). The level of 5-HT directly affects gastrointestinal function and fecal expulsion (Tuohongerbieke et al., 2024). The 5-HT3 and 5-HT4 receptors (5-HT3R and 5-HT4R), predominantly located on enteric neurons, mediate submucosal sensory neurons activation, leading to the release of acetylcholine and calcitonin gene-related peptides, which facilitate intestinal motility and defecation (Vlismas et al., 2024; Wan et al., 2023). Reduced levels of 5-HT and its receptors have been observed in STC patients, contributing to prolonged intestinal transit time and weakened motility (Wang et al., 2022; Wang et al., 2018). Our study corroborated these findings, showing that STC rats had decreased the expression of 5-HT, 5-HT3R, and 5-HT4R in the colonic tissue. Golden Bifid treatment significantly upregulated the expression of these markers, suggesting that it alleviates LOP-induced STC by enhancing 5-HT signaling and improving colonic motility. The mechanism underlying LOP-induced slow transit constipation extends beyond its role as an opioid receptor agonist with anti-diarrheal effects, involving profound pathological alterations in key regulatory molecules of intestinal function (Shen et al., 2024). Our study demonstrates that loperamide significantly reduced the expression levels of c-kit, 5-HT, 5-HT3R, and 5-HT4R in colonic tissue, consistent with findings by Zheng et al. (2021), who reported decreased c-kit expression in STC rat models (Zheng et al., 2021). The downregulation of c-kit, a marker protein for ICCs, directly reflects the compromised quantity and function of ICC, while inhibition of the 5-HT signaling pathways disrupts normal intestinal motility regulation mechanisms at multiple levels (Foong et al., 2020). This “cascade effect” may contribute to the persistent nature of loperamide-induced constipation symptoms, providing new insights into the molecular mechanisms of drug-induced constipation.

Whole-genome high-throughput RNA sequencing revealed that Golden Bifid modulates key signaling pathways involved in intestinal function. Compared to the LOP group, Golden Bifid-M treatment resulted in 1,998 differentially expressed transcripts, with 899 upregulated and 1,099 downregulated transcripts. KEGG pathway analysis identified significant enrichment in pathways such as MAPK, TNF signaling, and cell cycle regulation. The MAPK signaling pathway is involved in processes such as cell growth, differentiation, apoptosis, stress responses and inflammation. Its dysregulation has been implicated in the pathogenesis of STC, affecting smooth muscle function and colonic motility (Huizinga et al., 2021; Fung et al., 2019). IHC analysis further validated the RNA sequencing results, demonstrating increased phosphorylation of ERK in the MAPK signaling pathway in STC rats, along with upregulated expression of upstream regulators such as TGF-β and downstream effectors like Hspb1, Flnc, Hspa1b, Nfatc1, and Nfkb1. Golden Bifid treatment reversed these changes, reactivating the MAPK pathway and restoring normal signaling. These findings suggest that Golden Bifid may alleviate STC symptoms by modulating MAPK pathway, improving colonic motility, and promoting tissue repair.

STC is also associated with gut microbiota dysbiosis, characterized by alterations in microbial composition and reduced microbial diversity. Our 16S rDNA sequencing of feces revealed significant shifts in gut microbiota structure in STC rat, with increased *Firmicutes* and reduced *Bacteroidetes*, consistent with previous reports of microbial imbalances in constipation models (Yu et al., 2024). Golden Bifid treatment partially restored the composition of the gut microbiota, increasing microbial diversity and reversing dysbiosis. Specifically, Golden Bifid restored the abundance of *Patescibacteria*, a phylum significantly reduced in STC rats and linked to constipation progression.

At the genus level, Golden Bifid treatment reduced the abundance of harmful bacteria such as *Coprococcus*, which is associated with small intestinal bacterial overgrowth (Guo et al., 2024; Zhao et al., 2022), and promoted the expansion of beneficial bacteria such as *Turicibacter*, which has been reported to regulate neurotransmitter-related gut functions (Fung et al., 2019). These findings suggest that Golden Bifid improve STC symptoms by regulating the gut microbiota, enhancing the colonization of beneficial bacteria, and suppressing the growth of harmful bacteria.

Our study provides the first comprehensive multi-omic characterization of the mechanisms by which the clinically used probiotic formulation Golden Bifid alleviates slow transit constipation. Through integrating transcriptomic and microbiota analyses, we demonstrate that this formulation modulates key hots signaling pathways, (particularly MAPK and serotonergic signaling) and restores intestinal bacterial balance. These findings advance current understanding of probiotic mechanisms by moving beyond previously reported symptomatic relief to reveal specific molecular and microbial mediators underlying therapeutic efficacy. However, while our study demonstrates that this probiotic combination can alleviate constipation-related symptoms in a murine model, direct translation to humans use requires careful consideration. Differences between murine and human gut microbiomes and the changes in the human gut microbiome shifts and symptoms improvement in human subject with chronic constipation may take weeks to months, in contrast to the more rapid changes observed in mice. Furthermore, host-specific factors, such as microbial ecology, immune responses, and metabolic processes differ significantly between species, potentially influencing both the efficacy and safety profile of the probiotic intervention. Therefore, while our preclinical findings are promising and provide mechanistic insight, well-designed long-term clinical trials in humans are essential to evaluate the translational relevance of this probiotic combination before clinical application can be recommended.

## 5 Conclusion

This study demonstrates that Golden Bifid has significant potential as a novel therapeutic intervention for STC. It offers a promising, multifaceted approach to managing STC by targeting colonic motility, mucosal repair, and key signaling pathways. These findings provide a solid foundation for Golden Bifid as a therapeutic option of STC in humans, potentially advancing treatment strategies for STC yet challenging gastrointestinal disorder.

## Data Availability

The datasets presented in this study can be found in online repositories. The names of the repository/repositories and accession number(s) can be found below: https://www.ncbi.nlm.nih.gov/, GSE273280.
