# Supplementary material for: Therapeutic effects of three-strain probiotic combination on slow transit constipation: mechanistic insights into MAPK signaling pathway and gut microbiota restoration
Source: Front Pharmacol. 2025 Oct 20;16:1684442. doi: 10.3389/fphar.2025.1684442 (PMC12580145; doi:10.3389/fphar.2025.1684442)
Supplement: Supplementary file 1 [file Supplementaryfile1.doc]

**Table S1 Primer sequences used in qRT**-PCR

| Genes | Primer | Sequence (5’-3’) |
| --- | --- | --- |
| HSPA1B | Forward | AAGAATGCGCTCGAGTCCTA |
|  | Reverse | GATCTTGCCCTTGAGACCCT |
| PIK3CB | Forward | ACTGTGCAGACGAACCCATA |
|  | Reverse | GCAATCTCAGCTGCCTTCTC |
| FLNC | Forward | TCCACCGATGTGTCACTGAA |
|  | Reverse | CTCATTGCCTGAAGGAGCAC |
| CREB3L4 | Forward | CACCAAGGCAGAGGAGAGAA |
|  | Reverse | GCTCTCCAGCCCATCTATGT |
| GAPDH | Forward | TATGTCGTGGAGTCTACTGGCG |
|  | Reverse | ATGAGCCCTTCCACGATGC |

**Table S2 Enriched pathways between LOP group vs. Control group**

| No. | #Term | Database | ID |
| --- | --- | --- | --- |
| 1 | HIF-1 signaling pathway | KEGG PATHWAY | rno04066 |
| 2 | MAPK signaling pathway | KEGG PATHWAY | rno04010 |
| 3 | Resolution of Sister Chromatid Cohesion | Reactome | R-RNO-2500257 |
| 4 | Mitotic Prometaphase | Reactome | R-RNO-68877 |
| 5 | Regulation of TP53 Degradation | Reactome | R-RNO-6804757 |
| 6 | Regulation of TP53 Expression and Degradation | Reactome | R-RNO-6806003 |
| 7 | Influenza A | KEGG PATHWAY | rno05164 |
| 8 | TNF signaling pathway | KEGG PATHWAY | rno04668 |
| 9 | TP53 Regulates Transcription of Cell Cycle Genes | Reactome | R-RNO-6791312 |
| 10 | Polo-like kinase mediated events | Reactome | R-RNO-156711 |
| 11 | Estrogen signaling pathway | KEGG PATHWAY | rno04915 |
| 12 | Cyclin A/B1 associated events during G2/M transition | Reactome | R-RNO-69273 |
| 13 | Osteoclast differentiation | KEGG PATHWAY | rno04380 |
| 14 | Hepatitis C | KEGG PATHWAY | rno05160 |
| 15 | G2/M DNA replication checkpoint | Reactome | R-RNO-69478 |
| 16 | Aldosterone synthesis and secretion | KEGG PATHWAY | rno04925 |
| 17 | NOD-like receptor signaling pathway | KEGG PATHWAY | rno04621 |
| 18 | AGE-RAGE signaling pathway in diabetic complications | KEGG PATHWAY | rno04933 |
| 19 | RHO GTPases Activate Formins | Reactome | R-RNO-5663220 |
| 20 | Nitrogen metabolism | KEGG PATHWAY | rno00910 |
| 21 | Transcriptional misregulation in cancer | KEGG PATHWAY | rno05202 |
| 22 | Hepatitis B | KEGG PATHWAY | rno05161 |

**Table S3 Enriched pathways between LOP+LCBL-M group vs. LOP group**

| No. | #Term | Database | ID |
| --- | --- | --- | --- |
| 1 | Influenza A | KEGG PATHWAY | rno05164 |
| 2 | cGMP-PKG signaling pathway | KEGG PATHWAY | rno04022 |
| 3 | Regulation of actin cytoskeleton | KEGG PATHWAY | rno04810 |
| 4 | MAPK signaling pathway | KEGG PATHWAY | rno04010 |
| 5 | Fc gamma R-mediated phagocytosis | KEGG PATHWAY | rno04666 |
| 6 | Toxoplasmosis | KEGG PATHWAY | rno05145 |
| 7 | Pancreatic cancer | KEGG PATHWAY | rno05212 |
| 8 | Prostate cancer | KEGG PATHWAY | rno05215 |
| 9 | Estrogen signaling pathway | KEGG PATHWAY | rno04915 |
| 10 | HTLV-I infection | KEGG PATHWAY | rno05166 |
| 11 | SUMOylation of DNA replication proteins | Reactome | R-RNO-4615885 |
| 12 | Deubiquitination | Reactome | R-RNO-5688426 |
| 13 | Amoebiasis | KEGG PATHWAY | rno05146 |
| 14 | Measles | KEGG PATHWAY | rno05162 |
| 15 | Choline metabolism in cancer | KEGG PATHWAY | rno05231 |
| 16 | Transport of Mature Transcript to Cytoplasm | Reactome | R-RNO-72202 |
| 17 | Transport of Mature mRNA derived from an Intron-Containing Transcript | Reactome | R-RNO-159236 |
| 18 | AGE-RAGE signaling pathway in diabetic complications | KEGG PATHWAY | rno04933 |
| 19 | Ras signaling pathway | KEGG PATHWAY | rno04014 |
| 20 | Bacterial invasion of epithelial cells | KEGG PATHWAY | rno05100 |
| 21 | Adenosine P1 receptors | Reactome | R-RNO-417973 |
| 22 | Chagas disease (American trypanosomiasis) | KEGG PATHWAY | rno05142 |
| 23 | Non-small cell lung cancer | KEGG PATHWAY | rno05223 |
| 24 | Hepatitis B | KEGG PATHWAY | rno05161 |
| 25 | TNF signaling pathway | KEGG PATHWAY | rno04668 |
| 26 | Focal adhesion | KEGG PATHWAY | rno04510 |
| 27 | Small cell lung cancer | KEGG PATHWAY | rno05222 |
| 28 | Developmental Biology | Reactome | R-RNO-1266738 |
| 29 | EGFR tyrosine kinase inhibitor resistance | KEGG PATHWAY | rno01521 |
| 30 | Heterotrimeric G-protein signaling pathway-Gq alpha and Go alpha mediated pathway | PANTHER | P00027 |
| 31 | Inflammatory bowel disease (IBD) | KEGG PATHWAY | rno05321 |
| 32 | Longevity regulating pathway - multiple species | KEGG PATHWAY | rno04213 |
| 33 | Inflammation mediated by chemokine and cytokine signaling pathway | PANTHER | P00031 |
